# Growth Mindset Is Associated With Mastery Goals in Adulthood

**DOI:** 10.5964/ejop.11915

**Published:** 2024-08-30

**Authors:** Léa Tân Combette, Jean-Yves Rotgé, Liane Schmidt

**Affiliations:** 1 Institut du Cerveau (ICM) INSERM, Sorbonne Université, Paris, France; 2 Energie Jeunes Association, Paris, France; 3 Service de Psychiatrie d’Adultes, APHP Sorbonne Université, site Pitié-Salpêtrière, Paris, France; University of Palermo (UNIPA), Palermo, Italy

**Keywords:** fixed mindset, growth mindset, mastery goals, performance goals, adulthood

## Abstract

Growth mindsets, the belief that intelligence can grow with effort and training, have been associated with the adoption of mastery goals in children and adolescents. However, it is unknown whether these two factors are also correlated in adults. We conducted two online studies among three hundred participants to challenge this association. Results from (1) zero-order correlations, (2) structural equation modeling and (3) out-of-sample predictions converged on the finding that growth mindset was associated with the adoption of mastery goals in mathematics. This association generalized across different ways of measuring mindsets. Taken together the results provided new evidence for the idea that mindset and goal achievement are intrinsically related concepts, which remain associated across different life stages and generalize across populations.

Implicit theories of intelligence, such as the belief (mindset) that one’s intelligence can grow with effort and training, have been largely studied since 1970. Although mindset could be conceptualized as a continuum, two mindsets are usually opposed: a fixed mindset (i.e., thinking that intelligence is an innate immutable characteristic) and a growth mindset (i.e., thinking that intelligence is malleable). Adopting a growth mindset has been associated with better academic performance (Bettinger et al., 2018; Blackwell et al., 2007; Claro et al., 2016; Paunesku et al., 2015), enthusiasm toward challenges (Rege et al., 2021; Yeager et al., 2016; Yeager et al., 2019), grit (Duckworth et al., 2007) and well-being (Zeng et al., 2016).

Importantly, growth and fixed mindsets are closely related to the type of achievement goal a person adopts, which are usually measured in a school or academic context. Adopting achievement goals can involve mastery or performance, which further subdivide into four sub-components (i.e., mastery avoidance, mastery approach, performance avoidance and performance approach) according to Moller and Elliot (2006), or three sub-components (i.e., mastery, performance-approach and performance-avoidance) following Elliot and Church (1997). A student might avoid losing knowledge and skills (mastery avoidance), or may strive to improve knowledge (mastery approach). On the contrary, students, who adopt performance goals might either strive to avoid poor performances, such as for example a bad grade (performance avoidance), or they may be motivated by demonstrating knowledge and skills relative to their peers, such as being the best pupil of the class (performance approach) (Hulleman et al., 2010). Research has shown that adopting rather mastery goals compared over performance goals is usually associated with better school outcomes including higher levels of grit (Akin & Arslan, 2014) and better academic achievement (Alhadabi & Karpinski, 2020).

Implicit theories of intelligence (mindsets) and achievement goals are historically (Dweck & Yeager, 2019) and theoretically associated. From 1970 to 1980, a series of studies investigated students’ reactions to failure (Diener & Dweck, 1978; Dweck & Reppucci, 1973; Weiner & Kukla, 1970). These studies described, for the first time, two diametrically opposed reactions: hopelessness versus motivation to improve. This difference was initially attributed to differences in achievement goals (Elliott & Dweck, 1988) before being attributed to differences in mindsets (Dweck et al., 1995). Eventually, this research converged on the finding that achievement goals and mindsets are correlated (Blackwell et al., 2007; Cook et al., 2018; De Castella & Byrne, 2015; Robins & Pals, 2002; Yeager et al., 2016). However, studies from this research stream were conducted almost exclusively in children and adolescents and in the context of school and academia. It is thus completely unknown whether mindset and achievement goals are still correlated in an adult population and for everyday life use of knowledge and skills.

The main goals of this study were to test, (1) the correlation between mindset and achievement goal in adults, (2) how mindsets structurally linked to achievement goals in adults, and (3) how mindsets predicted achievement goals when measured by similar, but different mindset questionnaires.

To these aims we conducted two online studies in a total of three hundred adult participants. In both studies, mindsets and achievement goals were focused on one domain, math skills. This was done in order to restrict and contextualize the observed variables. Moreover, math skills are often associated with talent (Leslie et al., 2015). Participants will thus feel freer to declare that having a fixed mindset if it was the case. In sum, our approach was to establish the zero order correlations between mindset and achievement goals. Then we used structural equation modelling to gain more fine-grained and directional insight into the underlying structural relationships between mindsets (growth vs. fixed) and achievement goals (mastery vs. performance by avoidance vs approach). Lastly, we conducted an out-of-sample cross validation to test for how much mindsets predicted achievement goals across different populations (i.e., from participants in Study 1 to participants in Study 2), who were using different types of questionnaires to indicate their mindset about mathematic skills.

## Study 1

### Method

The study was conducted in accordance with the Declaration of Helsinki. The study protocol was approved by Sorbonne University’s independent ethics committee (CER-2021-006). All participants provided informed, fully-anonymized consent. The methodology and statistical analyses were pre-registered before any data collection (see Combette, 2022). We report all manipulations, measures, and exclusions in these studies. All materials, data and codes are accessible online (see Combette, 2024).

#### Participants

One hundred and fifty participants were recruited online. The sample size was determined based on the correlation between mindset and achievement goals of *r* = .23 reported by Yeager et al. (2016). Based on this value, the minimal sample size to attain a Type I error of .05 and a Type II error of .20 comprised 146 participants. This sample size also fell within a sample size range spanning between 56 and 147 participants for a structural equation model with maximally 6 latent variables and 15 measured variables following Sideridis et al. (2014). In adherence to the recommendations provided by the ethical committee, we limited the collection of socio-demographic variables to age and sex. Analysis of this data revealed a diverse adult population in our sample, spanning ages from 19 to 72 years (*M* = 34.86, *SE* = 0.87 years). Among the 150 participants, 113 identified as female, 35 as male, 1 as other, and 1 chose not to disclose this information. Furthermore, as our study was a component of a larger project that required monitoring participants’ familiarity with psychology and their occupation in scientific positions, we also inquired about their professional background. Participants were asked to specify whether they held a scientific position, worked in the field of psychology (as a researcher, psychologist, or psychology student), or were engaged in other fields. Responses to this question illuminated the diverse composition of our population, encompassing individuals employed across various fields and students (13% after excluding the psychology category).

#### Questionnaires

Maths Mindset was assessed by a 4-items mindset questionnaire building on the mindset scales reported by Dweck (1999). The scale was modified by contextualizing « intelligence » by « mathematic skills ». Two items assessed growth mindset and two items assessed fixed mindset. Participants responded on a five-point Likert response scale ranging from 1 (Totally disagree) to 5 (Totally agree). Mindset scores were computed by averaging responses to the two growth mindset items (growth mindset sub-score, *r* = .66) and responses to the two fixed mindset items (fixed mindset sub-score, *r* = .77). An intelligence mindset questionnaire was also completed by participants (see Combette et al., 2024).

Achievement goals were assessed by the 2x2 Achievement goal questionnaire—revised (Elliot & Murayama, 2008). The questionnaire built on the four-component model of achievement goals and comprised twelve questions (e.g., each one of the four achievement goal components was measured by three of the twelve questions) to which participants responded by using a five-point Likert scale ranging from 1 (Totally disagree) to 5 (Totally agree). Participants were instructed to focus on their achievement goals concerning mathematics skills in daily life. Scores were computed by averaging responses into four achievement goal sub-scores, which reflected the strength of approach (α = .84) and avoidance (α = .73) mastery goals, and approach (α = .89) and avoidance (α = .88) performance goals, respectively.

### Statistical Analyses

All analyses were conducted in R studio.

First, the association between the two mindset scores (i.e., growth and fixed mindset) and the four achievement goals (i.e., mastery approach, avoidance, performance approach, avoidance) was tested by conducting 2 x 4 zero order correlations. Correlations were calculated using the *cor.test* function of the *stats* package, and corrected for multiple comparisons using Bonferroni.

We then used structural equation modeling to obtain information about the structural relationship between the observed mindset and achievement goals variables (MacCallum & Austin, 2000; Weston & Gore, 2006). These analyses were added to the pre-registered correlational analyses in order to add directional insights.

Preliminary analyses testing the impact of participants’ sex on mindset showed that sex, *F*(2) = 1.45, *p* = .24 for fixed mindset and *F*(2) = 2.84, *p* = .06 for growth mindset, did not impact mindset, we thus decided not to include this variable in the following analyses.

#### Model Specification

**Figure 1:**
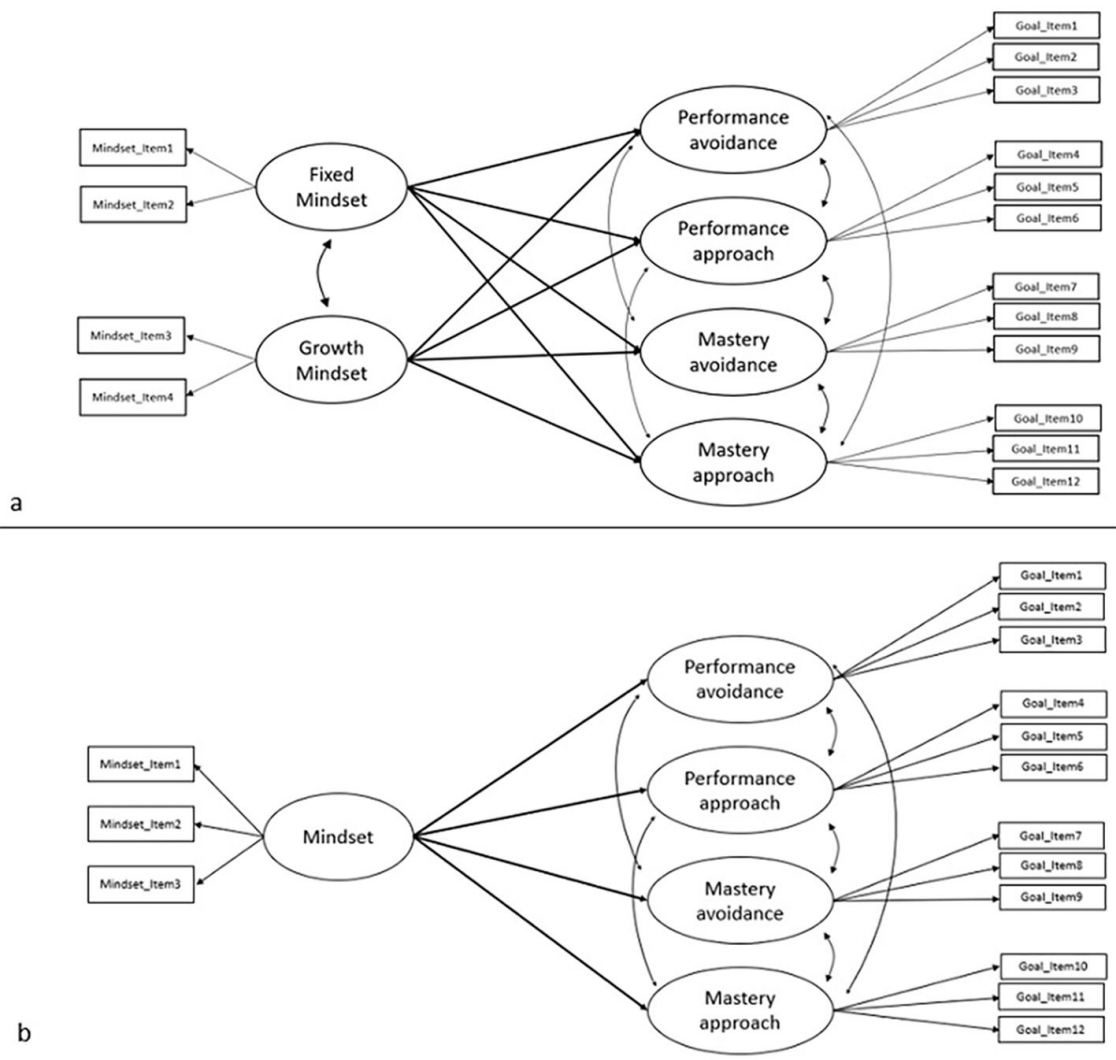
Schematic Overview of the Single-Level Structural Equation Models Fitted to Observed Mindset and Achievement Goal Variables *Note.* Two analogous models tested the association between mindsets and achievement goals. Black arrows indicate directional links between the mindset variables measured by a four (1a, Study 1) and a three-item (1b, Study 2) questionnaire (Dweck, 1999), and each type of achievement goal—mastery avoidance and approach, and performance avoidance and approach. In both studies, each type of achievement goal was scored by averaging the three corresponding items forming a twelve-item achievement goal questionnaire (Elliot & Murayama, 2008).

As shown in the path diagrams in Figure 1 the model assumed directional dependencies between mindsets and achievement goals based on prior theorizing of the conceptual links between these two observed variables (Blackwell et al., 2007). First, our model specified how factors were built. Fixed mindset was the average score across the two first items of the mindset questionnaire. Growth mindset was composed by averaging per participant the two last items of the mindset questionnaire. Mastery approach was composed of Items 1 to 3 of the achievement goal questionnaire. Mastery avoidance was composed of Items 4 to 6 of the achievement goal questionnaire. Performance approach was composed of Items 7 to 9 of the achievement goal questionnaire and Performance avoidance was composed of Items 10 to 12 of this questionnaire. Then, we regressed each achievement goals on each of the two mindset scores using single level path regressions.

#### Model Estimation

Single level path regressions and covariances were calculated using the *sem* function of *lavaan* package in R. Model estimation was based on maximum likelihood. We considered a threshold of *p* = .05 as significant for linear regressions. The model converged.

#### Model Fit

Two criteria were used to assess the fit of the model. The Comparative fit index (CFI) following Bentler (1990), and the Standardized Root Mean Square Residual (SRMR) following Browne and Cudeck (1992). The fit of the model was considered as good if the CFI was close to 1 (Finney, Pieper & Barron, 2004) and the SRMR was smaller than .08 (Hu & Bentler, 1999).

### Results

#### Zero-Order Correlations

Growth mindset about mathematics skills was positively correlated with approach mastery goals, *r* = .32, 95% CI = [0.171, 0.459], *p* = 4.62e-04, and avoidance mastery goals, *r* = .29, 95% CI = [0.137, 0.431], *p* = 2.0e-03, Bonferroni corrected. In other words, participants with a greater growth mindset were more likely to do mathematics in order to improve their mathematics skills or to avoid forgetting how to do mathematics (see Table 1).

**Table 1:** Zero-Order Correlations Between Mindsets and Achievement Goals

Mindset	Mastery App.	Mastery Av.	Performance App.	Performance Av.
**Fixed Mindset**	-.18*	-.14	-.01	.09
**Growth Mindset**	.32**	.29**	.11	.06

*Note*. Pearson’s regression coefficients are displayed within each panel.

** indicates significant correlation after Bonferroni correction (p < .05). * indicates significant correlation only before Bonferroni correction.

#### Structural Equation Modeling

**Figure 2:**
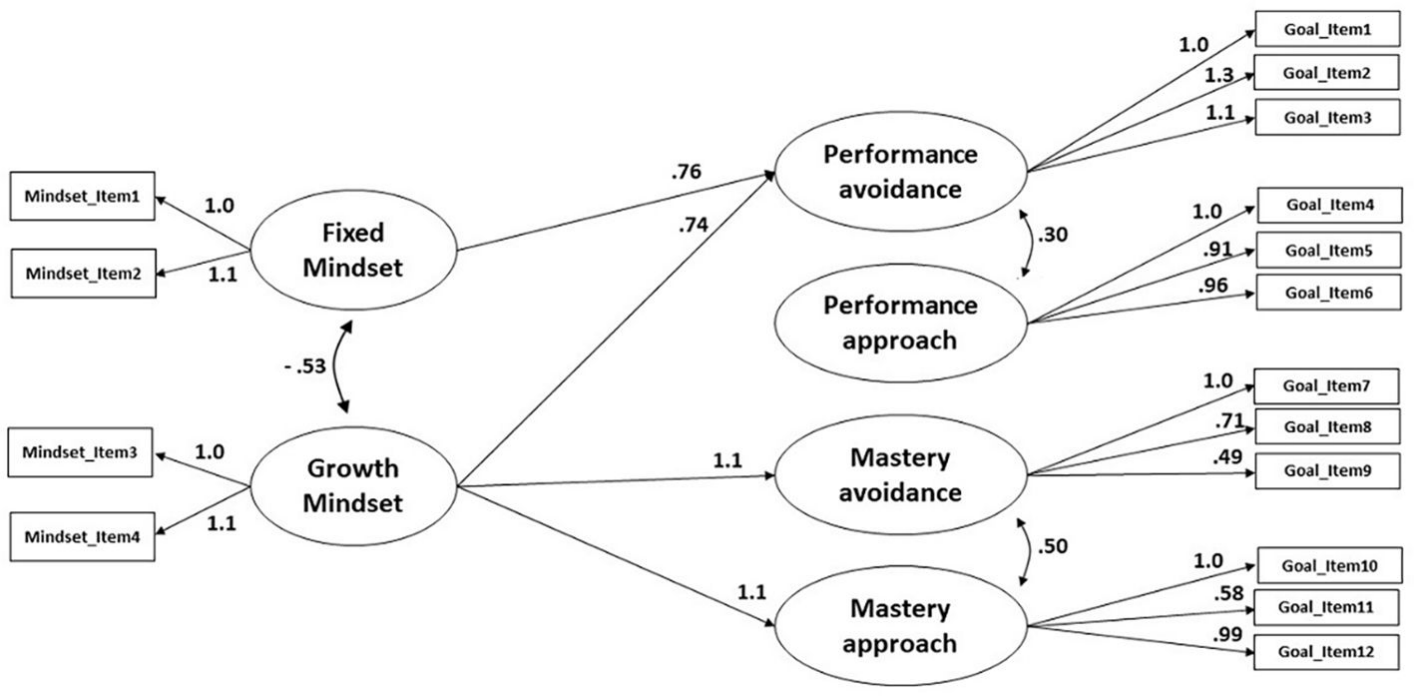
Results of SEM (Standardized Estimates for Statistically Significant Paths) for Growth Mindset and Achievement Goals *Note*. Black arrows indicated unidirectional links between observed variables, reciprocal arrows indicated covariances.

The fit of the model was good (CFI = .95 and SRMR = .06). As shown in Figure 2 the path regressions from growth mindset to mastery approach (β = 1.144, *SE*_mean_ = 0.438, *p* = .009) and to mastery avoidance (β = 1.100, *SE*_mean_ = 0.419, *p* = .009) were significant, which corresponded to the univariate zero order correlations observed for growth mindset and mastery goals. Importantly, when simultaneously controlling for these links between growth mindset and mastery goals, growth mindset (β = 0.741, *SE*_mean_ = 0.352, *p* = .035) and fixed mindset (β = 0.762, *SE*_mean_ = 0.350, *p* = .029) predicted the adoption of performance avoidance goals.

## Study 2

### Method

#### Participants

150 participants were recruited online. Similar to Study 1, the sample size was determined based on the correlation of *r* = .23 reported by Yeager et al. (2016), leading to a required minimum sample size of 146 participants. Similar to Study 1, our collection of socio-demographic variables for Study 2 was limited to age and sex. The sample for Study 2 exhibited diversity, with participants ranging in age from 20 to 70 years (*M* = 38.26, *SE* = 0.97 years). Out of the 150 participants, 130 identified as female, 19 as male, and 1 as other. Furthermore, an analysis of the question regarding professional background revealed a range of positions among participants. This included 9 students (excluding psychology students), 8 individuals in roles related to psychology, 101 with non-scientific positions, 31 with scientific positions, and 1 participant who chose not to disclose this information (see Table 2).

**Table 2:** Description of Study 1 and Study 2 Participants

Factor	Study 1	Study 2
Age	*M* = 34.8, *SD* = 0.87	*M* = 38.3, *SD* = 0.97
Sex	113 F / 35 M / 2 Other	130 F / 19 M / 1 Other
Education/Profession	20 Students (non-psychology) 11 in psychology 77 with non-scientific positions 41 with scientific positions 1 other	9 Students (non-psychology) 8 in psychology 101 with non-scientific positions 31 with scientific positions 1 other
Mindset	*M* = 3.91, *SD* = 1.00	FM: *M* = 2.06, *SD* = 0.88 GM: *M* = 3.89, *SD* = 0.95
Performance Avoidance	*M* = 2.78, *SD* = 0.96	*M* = 2.57, *SD* = 0.99
Performance Approach	*M* = 1.90, *SD* = 0.81	*M* = 1.71, *SD* = 0.71
Mastery Avoidance	*M* = 3.94, *SD* = 0.81	*M* = 3.93, *SD* = 0.80
Mastery Approach	*M* = 3.75, *SD* = 0.88	*M*m = 3.80, *SD* = 0.89

#### Questionnaires

Maths Mindset was assessed by the 3-items mindset questionnaire (Dweck, 1999). This scale was adapted by replacing “intelligence” by “mathematic skills”. Participants responded on a five-points scale ranging from 1 (Totally disagree) to 5 (Totally agree). All three items were framed within a fixed mindset position. Scores were computed by averaging responses to the three items and reversing it so that a score of 1 meant that participants hold a strong fixed mindset and a score of 5 meant that participants hold a strong growth mindset (α = .88). Achievement goals were measured with the exact same questionnaire used in Study 1.

### Analysis

Similar to Study 1, we calculated zero-order correlations and computed a structural equation modeling. Multiple correlations were also Bonferroni corrected leading to a significant threshold of *p* < 0.005. Additionally, we computed an out of sample prediction to test if the link between mindset and mastery goals, found in Study 1, generalized to a new group of participants. To this aim individual estimates of the impact of mindset on mastery goals were calculated using the lm function of the stats package in R. A new variable “predicted master goal” was then calculated from Study 1 individual betas and Study 2 individual mindset scores using the predict function from the car package in R. The predicted mastery goal scores where then correlated with measured mastery goal scores from Study 2 using the cor.test function from the stats package in R. This analysis was repeated three times to test three types of out-of-sample generalization: (1) for mastery approach goals, (2) for mastery avoidance goals (3) and for mastery goals, calculated by averaging mastery approach and mastery avoidance goals scores.

Analyses exploring the impact of participants’ sex on mindset showed that sex, *F*(2) = 1.45, *p* = .24 for fixed mindset and *F*(2) = 2.84, *p* = .06 for growth mindset, did not impact mindset, we thus decided not to include this variable in the following analyses.

### Results

#### Zero-Order Correlations

Math mindset (growth) was positively correlated with approach mastery goals, *r* = .23, 95% CI = [0.074, 0.377], *p* = 1.76e-02, Bonferroni corrected) and tendentially with avoidance mastery goals (*r* = .18, 95% CI = [0.021, 0.332], *p* = 10.6e-01, Bonferroni corrected. No significant correlation was found between mindset and performance goals (see Table 3).

**Table 3:** Zero-Order Correlations Between Mindsets and Achievement Goals

	Mastery App.	Mastery Av.	Performance App.	Performance Av.
**Mindset**	.23**	.18*	.01	.12

*Note.* Pearson’s regression coefficients are displayed within each panel.

** indicates significant correlation after Bonferroni correction (*p* < .05). * indicates significant correlation only before Bonferroni correction.

#### Structural Equation Modeling

**Figure 3:**
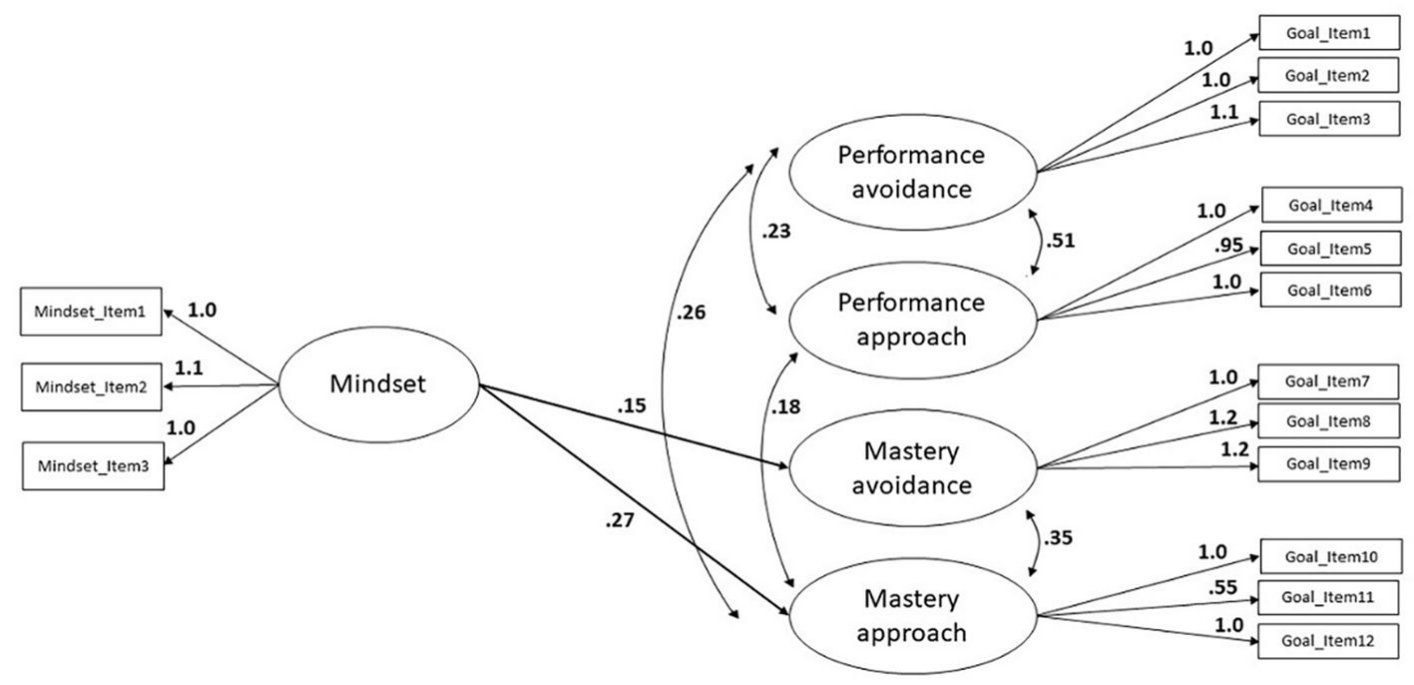
Results of SEM (Standardized Estimates for Statistically Significant Paths) for Growth Mindset and Achievement Goals *Note.* Black arrows indicated unidirectional links between observed variables, reciprocal arrows indicated covariances.

The analysis showed a good fit of the data (CFI = .94, SRMR = .07). As shown in Figure 3 mindset positively predicted mastery approach (β = 0.268, *SE*_mean_ = 0.092, *p* = .004) and mastery avoidance (β = 0.153, *SE*_mean_ = 0.066, *p* = .019). This finding indicated that participants with a growth mindset were more likely to do mathematics in order to improve their mathematics skills and to avoid forgetting how to do mathematics.

#### Out-of-Sample Prediction

**Figure 4:**
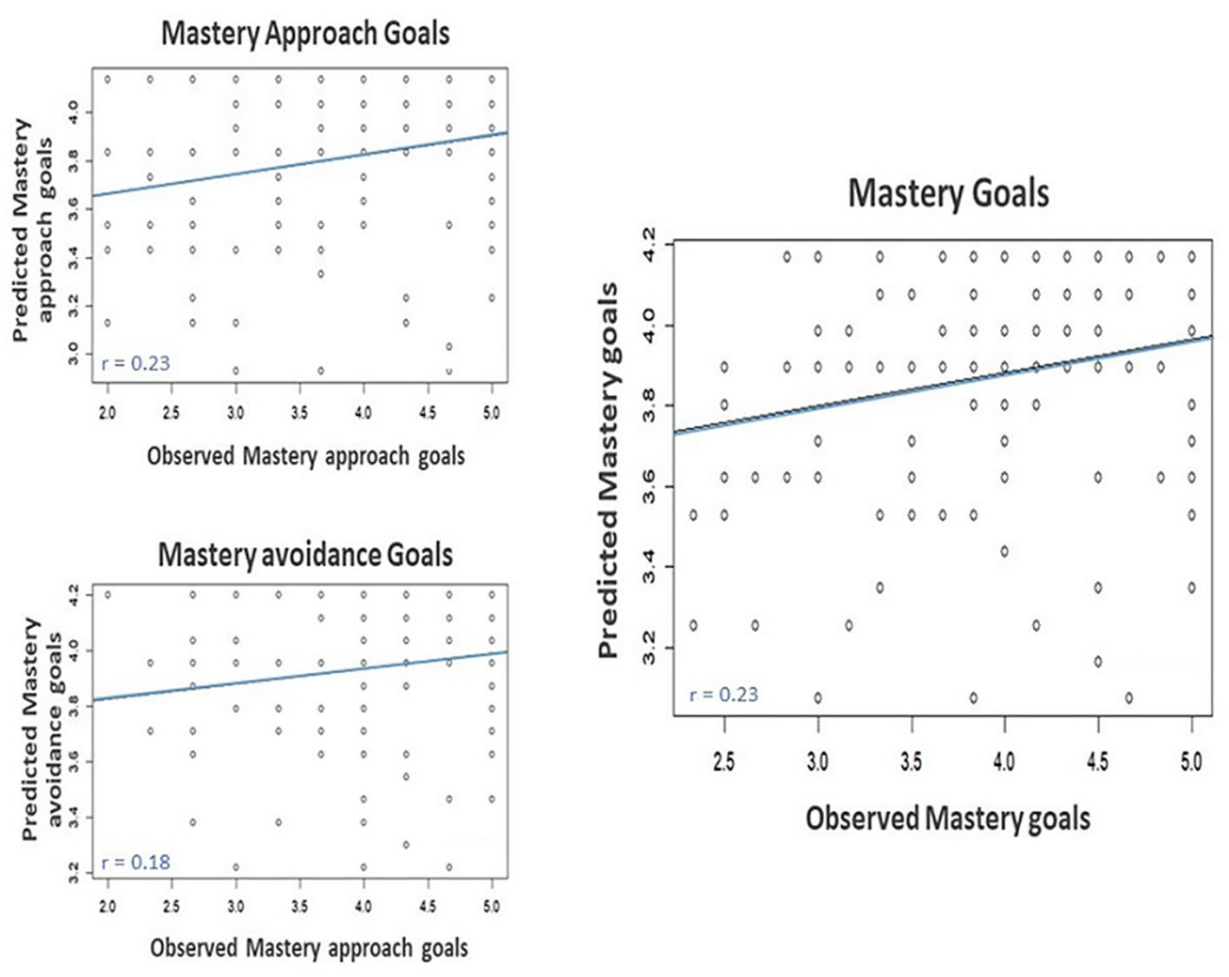
Correlation Between Predicted and Observed Mastery Goals for Out-Of-Sample Participants in Participants for Out-Of-Sample Participants From Study 2 When Considering the Weight of Mastery Goals in Participants in Study 1 *Note.* Mastery goals is calculated by averaging individuals’ mastery avoidance and mastery approach goals scores.

As shown in Figure 4, when correlating the predicted and observed mastery approach goals, *r* = .231, 95% CI = [0.074, 0.459], *p* = .013, Bonferroni corrected and predicted and observed combined mastery goals, *r* = .229, 95% CI = [0.072, 0.376], *p* = .377, Bonferroni corrected, were significant. Predicted and observed mastery avoidance goals, *r* = .181, 95% CI = [0.021, 0.332], *p* = .079, Bonferroni corrected, were tendentially correlated. These correlations provide evidence for a general role of growth mindsets for the prediction of mastery goals across adult populations and different ways of assessing mindsets.

## Discussion

In conclusion, here we provide convergent evidence across two studies and a total population of three hundred adult participants that growth mindsets about math skills predicted everyday math skill mastery goals. These associations were obtained while controlling for all other potential associations among observed mindset and goal achievement variables. Importantly, results from an out-of-sample cross-validation procedure showed that the link between mindset and mastery goals generalized across populations. These results show a robust association between mindset and mastery goal across populations and independently of the questionnaire used to assess mindsets. This finding validates the idea that mindset is a good predictor of mastery goals during adulthood.

The scope of our study was to extend the current knowledge about the association between mindset and achievement goals to an adult population. Much previous work has focused on the correlation between mindsets and achievement goals in children and adolescents in their school context (Blackwell et al., 2007; Cook et al., 2018; De Castella & Byrne, 2015; Robins & Pals, 2002; Yeager et al., 2016). Results from our two studies conducted among French adults confirmed that mindset and achievement goals are also associated in an adult population.

Moreover, this association was found when using two types of mindset questionnaires. A questionnaire measuring both, fixed mindset and growth mindset items, and a mindset questionnaire, which was composed of three items framed around fixed mindset (Dweck, 1999). Mindset questionnaires with only fixed mindset items are commonly used in the field of social psychology (Claro et al., 2016; Paunesku et al., 2015; Yeager et al., 2016). Indeed, using a small number of items related to the same idea could avoid participants’ boredom (Dweck et al., 1995). Moreover, fixed mindset items are known to be less influenced by the acquiescence bias (Claro et al., 2016), which consists in the propensity to agree a statement when actually in doubt. To rule out the possibility that these biases and confounds due to the type of mindset questionnaire influenced the association to achievement goals we conducted two studies measuring mindsets within both a growth and fixed mindset frame, and within a single, fixed mindset frame.

Significant positive correlations have been previously found between growth mindset and mastery goals (Blackwell et al., 2007; Cook et al., 2018; Robins & Pals, 2002) and between fixed mindset and avoidance performance goals (Robins & Pals, 2002; Yeager et al., 2016) and significant negative correlations have been found between fixed mindset and mastery goals (De Castella & Byrne, 2015). These correlations all convey the same idea that participants with a stronger growth mindset focus more on learning, whereas participants with a stronger fixed mindset focus more on performance and competition. Our results confirm that participants with greater growth mindsets are more likely to have mastery goals.

### New Research Questions and Limitations of Our Study

Our study raised new research questions and encouraged researchers to conduct follow-up studies. An important limitation of this study consists in the assessment of socio-demographic information, which was limited to self-reported sex and educational/professional background. Our study did not collect other factors such as household income, years of education, legal marital status, place of residence, or ethnic origin. Collecting this data and exploring its impact would be interesting to better understand the socioeconomic moderators of adults’ motivation. This is even more important given that studies conducted across secondary school students found that students from underprivileged backgrounds are more likely to adopt a fixed mindset and experience the impact of this mindset on their academic life (OECD, 2019). We, therefore, call for more research that assesses the socioeconomic sources for idiosyncrasies in mindsets and motivation.

Our correlational work is a prerequisite to identifying links that then can be targeted with interventions to test directionality or causality. Indeed, we do not know enough yet about what comes first achievement goals that lead to the adoption and maintenance of growth mindsets or growth mindsets that lead to preferring mastery over performance. This is important since wise interventions designed for adults might implement the mechanisms that incite for adoption of growth mindsets and translate them into more sustainable day-to-day motivation at home or the workplace.

Additionally, in our study, mindset, and achievement goals were measured by questionnaires. This approach was a necessary first step to test their association to identify the variables that could potentially be targeted by interventions. Future studies should therefore consider to now testing the impact of mindset interventions on achievement goals. Such a study would allow us to better understand the causality between mindset and achievement goals development in adults. This is important because adopting mastery goals has a positive impact on work and daily life. For example, mastery goals have been positively associated with strategic approaches to studying and negatively associated with surface learning in adult learners (Remedios & Richardson, 2013). Developing interventions helping participants to adopt both a growth mindset and mastery goals could have a beneficial impact on adults’ work and personal daily life.

## Data Availability

All materials, data and codes are accessible online (see Combette, 2024)
